# Integrating digital and interactive approaches in adolescent health literacy: a comprehensive review

**DOI:** 10.3389/fpubh.2024.1387874

**Published:** 2024-10-09

**Authors:** Stefania Mancone, Stefano Corrado, Beatrice Tosti, Giuseppe Spica, Pierluigi Diotaiuti

**Affiliations:** Department of Human, Social and Health Sciences, University of Cassino, Cassino, Lazio, Italy

**Keywords:** youth health literacy, digital education platforms, engaged learning, mental wellness literacy, social media in health, peer education models, innovative health tools, behavioral health outcomes

## Abstract

Adolescent health literacy is critical for navigating the complex landscape of modern healthcare and making informed decisions that influence long-term health outcomes. This comprehensive review synthesizes current research on integrating digital tools and interactive learning approaches to enhance health literacy among adolescents. We explore the use of digital technologies, such as mobile apps and virtual reality, which cater to the preferences of this tech-savvy generation, offering personalized and accessible health information. The effectiveness of interactive learning methodologies, including simulations and role-playing, is also examined, highlighting their potential to increase engagement and retention of health-related knowledge. We address the importance of mental health literacy and the role of social media and peer education in disseminating health information effectively. Our review identifies gaps in the current literature, particularly the need for studies that consider long-term outcomes and the impact of socioeconomic and cultural factors on health literacy initiatives. We propose a multidimensional approach to health literacy education that incorporates innovative technologies and interactive methods to meet the diverse needs of adolescents in various contexts. The findings suggest that an integrated approach, including digital and critical health literacy, is essential for developing comprehensive health education programs that are both informative and engaging for adolescents.

## 1 Introduction

Health literacy among adolescents is not just an emerging public health priority; it is a critical determinant of long-term health outcomes. As adolescents transition to adulthood, their ability to effectively access, process, and understand health information becomes crucial for making informed decisions that shape their health trajectories (1, 2). This capability directly influences their behavioral choices, such as dietary habits, physical activity, and interactions with healthcare systems (110–113). Adequate health literacy supports the development of self-care skills, effective management of chronic conditions, and preventive health behaviors, which are vital during this formative stage of life (3, 4). However, the extant literature reveals significant gaps in the health literacy of this demographic, pointing to a widespread inability to leverage health information for optimal health outcomes (5).

Current research on adolescent health literacy is fragmented and often constrained to narrow scopes that fail to capture the multifaceted nature of this field. Studies tend to concentrate on discrete populations or specific health behaviors, providing insights that are not easily generalizable to the broader adolescent population (6). There is a notable lack of integration in studies that consider how interconnected aspects of an adolescent’s life, such as mental health, social media influences, and educational environments, affect their health literacy (7, 8). This piecemeal approach fails to provide a holistic view of health literacy needs among adolescents. It does not adequately address how these needs vary across different socioeconomic, cultural, and environmental contexts. The lack of comprehensive models that encompass the full spectrum of health literacy, including digital and critical health literacy, further compounds these gaps.

This narrative review aims to synthesize the current research landscape on adolescent health literacy with a broader lens, addressing the noted research gaps by focusing on the comprehensive needs of this group. We aim to explore the integration of digital technologies, which play a pivotal role in today’s adolescent experience, and examine how these tools can enhance traditional health literacy approaches. The review will also assess the effectiveness of interactive learning methodologies that engage adolescents more deeply by connecting health information to their everyday lives through simulations, role-playing, and peer education. By doing so, we intend to identify and highlight successful strategies for enhancing health literacy among adolescents and discuss the challenges and limitations faced in these endeavors. We will go into the vital topic of mental health literacy, arguing that addressing adolescents’ comprehensive health needs requires a more integrated approach to health literacy, with mental health as a major component. Through this review, we aim to contribute to the existing body of knowledge by offering a deeper understanding of how different aspects of health literacy can be effectively integrated into age-appropriate and culturally sensitive interventions. We will also outline future research directions that could potentially fill the gaps in our understanding of adolescent health literacy, with the ultimate goal of fostering a generation of health-literate individuals equipped to make informed decisions about their health and well-being.

## 2 Study selection and evaluation methodology

### 2.1 Selection of studies

The methodology for selecting studies for this narrative review was rigorously designed to ensure relevance and comprehensive coverage of the topic. We identified the most pertinent health literacy research databases, including PubMed, Scopus, Web of Science, and PsycINFO. Our search strategy was crafted to capture a wide range of studies by employing a combination of keywords and MeSH terms such as “health literacy,” “adolescents,” “digital literacy,” “interactive learning,” and “mental health literacy. The search was extended to include grey literature and conference proceedings to ensure comprehensive coverage of unpublished studies and ongoing research efforts. We employed advanced search strategies that involved Boolean operators to refine and focus the search results. For instance, combinations such as “adolescents AND health literacy AND digital tools” were used to pinpoint studies that specifically addressed these intersecting themes.

The inclusion criteria were meticulously defined to focus on studies published in English from 2005 to 2024 to capture the most contemporary and relevant data. We included quantitative and qualitative research studies that focused specifically on the health literacy of adolescents. To ensure a comprehensive review, we considered studies that provided empirical data directly related to health literacy interventions, outcomes, and adolescent engagement with health literacy initiatives.

Exclusion criteria were applied to filter out studies that did not meet our thematic or demographic focus. These included studies on populations outside the adolescent age range (10–19 years), non-English language studies, and those that did not offer direct insight into health literacy, such as studies focusing solely on general education or unrelated health interventions.

### 2.2 Evaluation of methodological quality

We adopted a robust methodological quality assessment tool to ensure the credibility and reliability of the findings synthesized in this review. Each selected study was evaluated based on several criteria: clarity of research objectives, soundness of study design, appropriateness of the analytical methods, and the relevance and rigor of the conclusions drawn.

Two independent reviewers conducted the evaluations to minimize bias and enhance the objectivity of the analysis. Any discrepancies between reviewers were resolved through discussion or, if needed, consultation with a third expert reviewer. This dual-review process ensured that only studies meeting high standards of methodological rigor were included in our synthesis.

### 2.3 Data analysis and synthesis

The data extraction and synthesis process were tailored to align with the review’s objectives. For each study, the critical information such as study design, population characteristics, interventions, and outcomes were measured, and these findings were systematically recorded. We employed a thematic synthesis approach, where data were categorized and analyzed according to the themes relevant to our review objectives, such as the effectiveness of digital tools, the impact of interactive learning, and approaches to mental health literacy.

The synthesis involved an iterative process of identifying patterns, relationships, and gaps across the selected studies. This allowed us to construct a narrative that addresses the initial research questions and highlights areas requiring further exploration. By integrating findings from diverse studies, we aimed to provide a comprehensive overview of the current state of adolescent health literacy, emphasizing innovative strategies and critical areas for future research. Table 1 provides a detailed summary of selected studies that have significantly contributed to our understanding of adolescent health literacy. These studies focus on various innovative approaches, ranging from the integration of digital technologies to interactive learning methods. This summary table highlights the critical aspects of each study, including methodologies, populations studied, and principal findings, offering a comprehensive glance at the diverse strategies employed to enhance health literacy among adolescents. Each study is also linked to its corresponding citation in the reference section for clarity and further reference.

**Table 1:** Studies summary by thematic sections.

Reference	Thematic section	Study focus	Study type	Population	Intervention	Key findings
Jacobs et al. (27)	Use of digital technologies	Systematic review of eHealth interventions to improve health literacy	Systematic review	General population	eHealth Interventions	eHealth interventions have the potential to improve health literacy across various populations.
Grist et al. (43)	Use of digital Technologies	Systematic review of mental health mobile apps for preadolescents and adolescents	Systematic review	Preadolescents and adolescents	Mental health apps	Apps show promise in improving mental health literacy.
Rose et al. (29)	Use of digital technologies	Systematic review of digital interventions for diet and physical activity among adolescents	Systematic review	Adolescents	Digital interventions	Effective in improving diet and physical activity behaviors.
Badawy et al. (42)	Use of digital technologies	Systematic review of text messaging and mobile apps to improve adherence in adolescents with chronic health conditions	Systematic review	Adolescents with chronic conditions	Text messaging and apps	Text messaging and apps improve adherence.
Tabong et al. (41)	Use of digital technologies	Acceptability of school-based sexual and reproductive health services delivered by trained psychologists in Ghana	Qualitative study	Ghanaian adolescents	School-based services	School-based services are feasible and acceptable.
Huang et al. (47)	Use of digital technologies	Study on augmented reality (AR) and virtual reality (VR) in science education	Exploratory study	Science students	AR/VR applications	AR/VR improves science knowledge retention.
Huang et al. (46)	Use of digital technologies	Comparison of AR and VR in dentistry education	Comparative study	Dental students	AR/VR applications	AR/VR is effective in dental education.
Wadham et al. (31)	Use of digital technologies	Systematic review of new digital media interventions for sexual health promotion among young people	Systematic review	Young people	Digital media interventions	Effective in promoting sexual health among young people.
Pérez et al. (44)	Use of digital technologies	Review of mobile and web-based apps for self-management in young people with chronic illness	Systematic review	Young people with chronic illness	Self-management apps	Apps support self-management in chronic illness.
Jeminiwa et al. (28)	Use of digital technologies	Theoretical framework development for evaluating the quality of mHealth apps for adolescent users	Theoretical framework	Adolescent mHealth users	mHealth app evaluation	Framework assists in evaluating mHealth apps.
Tornivuori et al. (30)	Use of digital technologies	Systematic review on digital services supporting chronically ill adolescents during care transition	Systematic review	Chronically ill adolescents	Digital services	Supports transition of care for chronically ill adolescents.
Fernández et al. (26)	Use of digital technologies	Systematic review of sex education programs	Systematic review	General population	Sex education programs	Highlights best practices in sex education.
Hammond et al. (32)	Use of digital technologies	Digital storytelling for mental health, identity, and education for adolescents in state care	Qualitative study	Adolescents in state care	Digital storytelling	Enhances mental health, identity, and education.
Meherali et al. (33)	Use of digital technologies	Systematic review of digital literacy empowerment for adolescent girls in low- and middle-income countries	Systematic review	Adolescent girls in low- and middle-income countries (LMICs)	Digital literacy programs	Digital literacy empowers adolescent girls in LMICs.
König et al. (39)	Use of digital technologies	Development and evaluation of an e-learning course promoting digital health literacy in school-age children	Intervention study	School-age children	E-learning course	E-learning is effective in promoting digital health literacy.
Jamri et al. (38)	Use of digital technologies	Systematic review of social media interventions to improve nutrition behavior among adolescents in Asia	Systematic review	Asian adolescents	Social media interventions	Improves nutrition behaviors among adolescents.
Lytvynova and Soroko (49)	Use of digital technologies	Interaction in educational environments using virtual and AR	Exploratory study	General Population	AR/VR Applications	Enhances interaction in educational settings.
Alowais et al. (37)	Use of digital technologies	Scoping review of digital literacy in undergraduate pharmacy education	Scoping review	Pharmacy students	Digital literacy programs	Highlights the importance of digital literacy in pharmacy education.
Vamos et al. (36)	Use of digital technologies	Health literacy needs and preferences for a technology-based intervention to improve college students’ sexual and reproductive health	Survey study	College students	Technology-based intervention	Technology-based interventions are preferred for improving sexual and reproductive health literacy among college students.
Lee et al. (48)	Use of digital technologies	Supporting youth mental health through co-designing a virtual reality experience	Qualitative study	Youth	Virtual reality experience	Co-designing virtual reality experiences can effectively support youth mental health.
Rezaee et al. (45)	Use of digital technologies	Design and usability evaluation of a mobile application for self-care among Iranian adolescents	Intervention study	Iranian adolescents	Mobile application	Mobile applications can be effective tools for self-care management among adolescents.
Saudagar et al. (50)	Use of digital technologies	Comprehensive analysis of augmented reality (AR) and virtual reality (VR) integration in medical education for diverse abilities	Comprehensive analysis	Medical students with diverse abilities	AR/VR applications	AR/VR integration is beneficial in medical education.
Naef et al. (40)	Use of digital technologies	Qualitative study on adolescents with type 1 diabetes and their perspectives on digital health interventions	Qualitative study	Adolescents with type 1 diabetes	Digital Health Interventions	Positive reception of digital interventions by adolescents.
Raeside et al. (34)	Use of digital technologies	Stakeholder insights on adolescent digital health prevention programs in Australia	Qualitative study	Australian adolescents	Digital health programs	Identifies key factors for successful implementation.
Harris et al. (58)	Interactive learning methodologies	Technology’s role in diabetes management among teenagers	Qualitative study	Teenagers with diabetes	Diabetes management technologies	Technology aids in managing diabetes in teenagers.
Mason-Jones et al. (56)	Interactive learning methodologies	Review of school-based interventions for preventing human immunodeficiency virus (HIV), sexually transmitted infections (STIs), and pregnancy in adolescents	Systematic review	Adolescents	School-based interventions	School-based interventions are effective in preventing HIV/STIs.
Patton et al. (61)	Interactive learning methodologies	A Lancet commission report on adolescent health and well-being	Commission report	Adolescents	Health and well-being interventions	Comprehensive overview of adolescent health and wellbeing.
Haruna et al. (52)	Interactive learning methodologies	Improving sexual health education through game-based learning and gamification	Intervention Study	Adolescent Students	Game-based Learning	Game-based learning improves sexual health education.
Guo et al. (7)	Interactive learning methodologies	Quality of health literacy instruments used in children and adolescents: a systematic review	Systematic review	Children and adolescents	Health literacy instruments	The study evaluated the quality of health literacy instruments used with children and adolescents, highlighting the need for improved measures.
Rønning and Bjørkly (54)	Interactive learning methodologies	Use of clinical role-play and reflection in therapeutic communication skills learning	Integrative review	Mental health students	Role-play and reflection	Role-play is effective in learning therapeutic communication.
Mallia et al. (59)	Interactive learning methodologies	Media literacy intervention for performance and appearance enhancing substances (PAES) use in sport science students	Intervention study	Sports science students	Media literacy intervention	Media literacy reduces PAES use in sports.
Corcoran et al. (55)	Interactive learning methodologies	Integrative review of adolescents’ perceptions of sexual health education programs	Integrative review	Adolescents	Sexual health education	Programs are positively perceived by adolescents.
Citrawathi et al. (51)	Interactive learning methodologies	Effect of problem-based adolescent reproductive health module on students’ life skills	Intervention study	Adolescent students	Reproductive health module	Improves life skills and attitudes toward reproductive health.
Linggi et al. (62)	Interactive learning methodologies	The role of health literacy on adolescent smoking behavior in senior high school students	Survey study	High school students	Health literacy education	Health literacy was found to play a significant role in influencing adolescent smoking behavior, with higher literacy linked to reduced smoking rates.
Joseph and Fleary (2)	Interactive learning methodologies	“The way you interpret health”: Adolescent definitions and perceptions of health literacy	Qualitative study	Adolescents	Health literacy exploration	The study explored adolescents’ definitions and perceptions of health literacy, revealing that their understanding is influenced by personal and cultural factors.
Haruna et al. (53)	Interactive learning methodologies	Gamifying sexual education for adolescents in a low-tech setting: Quasi-experimental design study	Quasi-experimental design	Adolescents in low-tech settings	Gamified sexual education	Gamification in sexual education was found to be effective in engaging adolescents and improving their understanding of sexual health in low-tech settings.
Bektaş et al. (63)	Interactive learning methodologies	Predicting healthy lifestyle behaviors based on health literacy and self-efficacy in Turkish adolescents	Predictive study	Turkish adolescents	Health literacy assessment	Health literacy predicts healthy lifestyle behaviors.
Nash and Arora (60)	Interactive Learning Methodologies	Systematic review of health literacy interventions for Aboriginal and Torres Strait Islander peoples	Systematic review	Indigenous populations	Health literacy interventions	Improves health literacy among Indigenous populations.
Ribeiro et al. (57)	Interactive learning methodologies	Playful educational interventions in children and adolescents’ health literacy: a systematic review	Systematic review	Children and adolescents	Playful educational interventions	Playful educational interventions were found to be effective in improving health literacy among children and adolescents.
Burns and Rapee (65)	Mental health literacy	Study on adolescent mental health literacy and help-seeking behaviors	Survey study	Adolescents	Mental health literacy assessment	Adolescents have limited mental health literacy.
Wei et al. (71)	Mental health literacy	Effectiveness of school mental health literacy programs	Randomized controlled trial (RCT)	Adolescents	School mental health programs	Programs effectively address mental health literacy.
Skre et al. (75)	Mental health literacy	Effectiveness of a school intervention for mental health literacy	Survey study	Adolescents	School mental health programs	The intervention showed positive effects on mental health literacy.
Bjørnsen et al. (64)	Mental health literacy	Relationship between positive mental health literacy and well-being among adolescents	Survey study	adolescents	School mental health programs	Positive mental health literacy correlates with well-being.
Attygalle et al. (66)	Mental health literacy	Mental health literacy in adolescents: Ability to recognize problems, helpful interventions, and outcomes	Survey study	Adolescents	Mental health literacy education	The study highlighted gaps in adolescents’ ability to recognize and address mental health problems.
Mansfield et al. (8)	Mental health literacy	Systematic literature review of existing conceptualization and measurement of mental health literacy in adolescent research	Systematic review	Adolescents	Mental health literacy measures	The study identified challenges and inconsistencies in how mental health literacy is conceptualized and measured.
Simkiss et al. (74)	Mental health literacy	Improving mental health literacy in high school children across Wales: A protocol for a RCT	RCT	High school students	Mental health literacy program	The protocol outlines a mental health literacy program aimed at improving understanding and reducing stigma.
Amudhan et al. (72)	Mental health literacy	Project scaling up of mental health in schools (SUMS): Design and methods for a pragmatic, cluster-randomized waitlist-controlled trial on integrated school mental health intervention for adolescents	Cluster randomized controlled trial (waitlist controlled)	Adolescents in schools	Integrated school mental health intervention	The study focuses on scaling up mental health interventions in schools through an integrated approach.
Hassen et al. (67)	Mental health literacy	Impact of social media on mental health literacy among adolescents	RCT	Adolescents	Social media literacy	Social media improves mental health literacy among adolescents.
Panza et al. (68)	Mental health literacy	Development and evaluation of peer-based mental health literacy intervention in adolescent athletes	Brief report	Adolescent athletes	Peer education	Peer education is effective in improving mental health literacy.
Berger et al. (73)	Mental health literacy	Long-term benefits of school mental health interventions	Survey study	Norwegian adolescents	School mental health programs	Non-randomized trials show positive effects on mental health literacy.
Sokolová (69)	Mental health literacy	Brief report on mental health literacy and professional help-seeking in Slovakia	Survey study	Slovak adolescents	School mental health programs	Parent–child relationship mediates mental health literacy.
Wang et al. (70)	Mental health literacy	Mediating effect of parent–child relationship on mental health literacy transmission	Systematic review	Chinese parents and children	Parent–child relationship program	Parent–child relationship plays a significant role in mental health literacy transmission.
Topping et al. (76)	Mental health literacy	Assessing the Irish Football Association’s “Ahead of the Game” coach education training program on raising mental health literacy in youth football coaches	Evaluation study	Youth football coaches	Coach education training program	The program effectively improved mental health literacy among youth football coaches, positively impacting their knowledge and attitudes.
Smith and Petosa (87)	Impact of social media and peer education	Peer mentoring method for physical activity behavior change among adolescents	RCT	High school students	Peer-led intervention	Peer mentoring is effective for behavior change.
Moorhead et al. (80)	Impact of social media and peer education	Review of social media’s uses, benefits, and limitations for health communication	Systematic review	General population	Health information seeking	Social media is effective for health purposes.
Li et al. (79)	Impact of social media and peer education	Evaluation of a web-based social network game for enhancing mental health literacy	RCT	High school students	Peer education	Social media strategy increases mental health literacy.
Sudo and Kuroda (94)	Impact of social media and peer education	Impact of media exposure and health literacy on adolescent smoking susceptibility	Survey study	High school students	Educational interventions	Media literacy influences smoking behaviors.
Pisani et al. (85)	Impact of social media and Peer Education	Pilot study on text messaging in a school-based substance use prevention program	Pilot study	High school students	Text messaging program	Text messaging supports substance use prevention.
Halsall et al. (78)	Impact of social media and peer education	Evaluation of a social media strategy to promote mental health literacy in youth	Survey study	Adolescents	Social media strategy	Social media can enhance health literacy education.
Weybright et al. (95)	Impact of social media and peer education	Positive outcomes of the “Teens as Teachers” program for promoting healthy nutrition in adolescents	Pilot study	High school students	Peer education	Peer education promotes healthy nutrition.
Russell et al. (91)	Impact of social media and peer education	Feasibility of a peer education project to improve mental health literacy in the UK	Feasibility study	Young people	Peer education	Peer education is feasible and effective in mental health literacy.
Widnall et al. (97)	Impact of social media and peer education	Process evaluation of a peer education project to improve mental health literacy	Process evaluation	Secondary school students	Web-based game	Peer education mechanisms are complex and multifaceted.
Taba et al. (35)	Impact of social media and peer education	Cross-sectional study on adolescents’ self-efficacy and digital health literacy	Cross-sectional Study	School children	Media literacy education	Self-efficacy is linked to digital health literacy.
Scull et al. (92)	Impact of social media and peer education	Media literacy education approach to high school sexual health education	Intervention study	General population	Smoking prevention program	Media literacy is effective in sexual health education.
Booth et al. (83)	Impact of social media and Peer Education	Comparative study of peer- vs. adult-led health promotion delivery in schools	Comparative study	Female adolescents	Media literacy program	Video-assisted education improves infectious disease literacy.
Ameneh et al. (82)	Impact of social media and peer education	Peer-led diabetes intervention in female adolescents in schools	RCT	High school students	Peer-led intervention	Peer-led interventions improve diabetes management.
Islam et al. (84)	Impact of social media and peer education	Feasibility and acceptability of virtual adolescent diabetes prevention program	Qualitative study	High school students	Virtual program	Virtual programs are feasible and acceptable.
Chuene (89)	Impact of social media and peer education	Challenges faced by peer educators in health promotion activities at higher education institutions	Qualitative study	High school students	Peer education challenges	Peer educators encounter implementation challenges.
Chen and Wang (77)	Impact of social media and peer education	Systematic review of social media use for health purposes	Systematic review	School-based adolescents	Peer education program	Health literacy influences health information seeking.
Niu et al. (81)	Impact of social media and peer education	Associations between health literacy, social media use, and health information-seeking intentions in China	Survey study	Adolescent females	Peer education	Peer educators face challenges in health promotion.
Widnall et al. (96)	Impact of social media and peer education	Mechanisms of school-based peer education interventions to improve health literacy	Realist review	Adolescents	Social media strategy	Peer-led delivery is as effective as adult-led delivery.
Kim Kim, S (90)	Impact of social media and peer education	Using intervention mapping to develop a media literacy-based smoking prevention program for female adolescents	Intervention development	Female adolescents	Media literacy-based smoking prevention program	The study utilized intervention mapping to develop a media literacy-based program, which effectively raised awareness and reduced smoking intentions.
Ratnawati et al. (86)	Impact of social media and peer education	Phenomenological study on HIV/AIDS prevention behavior among adolescent peer educators in Indonesia	Phenomenological study	Adolescents with diabetes	Peer mentoring	Text messaging supports substance use prevention.
Shoghli et al. (93)	Impact of social media and peer education	Effect of peer-to-peer education on health literacy and adherence to coronavirus disease 2019 (COVID-19) protocols	Pilot study	Secondary school students	Peer education	Peer education improves health literacy and adherence.
Zhang et al. (109)	Impact of social media and peer education	Video-assisted health education followed by peer education to improve COVID-19 literacy among school children	RCT	Children and adolescents	Peer education	Prevention programs reduce smoking susceptibility.

## 3 Theoretical framework

### 3.1 Health literacy model

The concept of health literacy extends beyond the essential ability to read and comprehend medical literature; it encompasses a range of skills including accessing, understanding, evaluating, and using health information to make appropriate health decisions (9). To structure our analysis, we adopt Nutbeam’s model of health literacy, which categorizes health literacy into three distinct levels: functional, interactive, and critical (10–12).

- “Functional Health Literacy” refers to basic skills in reading and writing that are necessary to function effectively in everyday situations involving health.
- “Interactive Health Literacy” involves more advanced cognitive and social skills that enable individuals to actively participate in their healthcare, extract information from different communication channels, and derive meaning from complex forms of communication.
- “Critical Health Literacy” encompasses advanced cognitive skills that allow individuals to critically analyze information, and use this information to exert greater control over life events and situations.

This model is the backbone for our review, providing a comprehensive framework to assess how adolescents interpret and act upon health messages and education in varying contexts.

In addition to Nutbeam’s model, our review integrates broader health literacy, including civic and cultural literacy. These frameworks are particularly pertinent, encompassing the social and cultural competencies required to navigate the increasingly global and digitally connected health information environment. Civic literacy, for instance, enhances understanding of one’s rights and responsibilities in health contexts, a crucial aspect as adolescents engage more frequently with health information through digital platforms (13, 14). Cultural literacy facilitates a deeper understanding of how health behaviors and communication are influenced by cultural norms, which is vital for designing culturally sensitive health literacy interventions (15, 16).

### 3.2 Linking with existing theories

To deepen our understanding of health literacy among adolescents, we must integrate established health behavior theories that explain why and how individuals make health-related decisions. Two pivotal theories, the “Health Belief Model (HBM)” and the “Theory of Planned Behavior (TPB),” are particularly relevant in framing our discussion:

- Health Belief Model (HBM): This model predicts that individuals are more likely to take action toward a health-related behavior if they perceive a personal threat of a disease or condition (perceived severity and susceptibility), believe that a specific action available to them would reduce this threat (perceived benefits), and perceive few negative aspects to take action (perceived barriers). Cues to action and self-efficacy are the critical components of this model (17–19). In the context of health literacy, understanding how adolescents perceive these elements can illuminate the pathways through which health literacy affects health actions.
- Theory of Planned Behavior (TPB): TPB extends the understanding of health behavior by explaining that an individual’s behavior is directly influenced by their intention to perform the behavior, which in turn is influenced by their attitudes toward the behavior, subjective norms, and perceived behavioral control (20–22). Regarding health literacy, adolescents’ engagement in health-literate behaviors can be explored by examining their attitudes toward health information, the influence of peers and social norms, and their confidence in their ability to obtain, understand, and use health information.

By integrating these theories with the health literacy model, our review will explore not only the direct impact of health literacy skills on adolescent health outcomes but also the sociopsychological processes that underpin these effects (23–25). This theoretical framework allows for a multidimensional analysis of the factors contributing to effective health literacy interventions. It highlights potential areas for targeted improvements in adolescent health education programs.

## 4 Results of the literature

### 4.1 Use of digital technologies

The integration of digital technologies in health literacy initiatives has shown significant promise in enhancing the accessibility and effectiveness of health education for adolescents (26–31). Studies consistently highlight that digital platforms, such as mobile apps, websites, and online games, cater effectively to the preferences of a tech-savvy generation (32–36). These tools not only facilitate the dissemination of health information but also enable interactive and personalized learning experiences (37–41). For instance, mobile health applications have been found to improve adolescents’ engagement with health monitoring and management tasks, particularly in areas such as diabetes self-care and mental health tracking (42–45). Virtual and augmented reality (AR) applications are also emerging as potent tools for simulating real-life scenarios, thereby allowing adolescents to practice decision-making in a risk-free environment (46–50).

### 4.2 Interactive learning methodologies

Interactive learning methodologies, including problem-based learning, role-playing, and simulation-based activities, have been extensively documented for their efficacy in improving health literacy among adolescents. Such methodologies encourage active learning and critical thinking, which are essential for the practical application of health-related knowledge (51–54). For example, role-playing scenarios that mimic doctor-patient interactions can enhance understanding and retention of information related to sexual health and substance abuse prevention (55–57). Interactive workshops that involve peer discussions and collaborative problem-solving have been shown to effectively increase knowledge and change attitudes toward complex health issues such as nutrition and physical activity (58–60).

Interactive learning methods, such as problem-based learning and peer-led discussions, enhance health literacy and promote broader educational outcomes. These methodologies support critical thinking and problem-solving skills, which are essential in the holistic development of adolescents (7, 61). By engaging in these active learning processes, adolescents not only learn to apply health-related knowledge in practical contexts but also develop key skills that aid their academic and personal growth (2, 62). Such dynamic educational experiences prepare adolescents to face complex real-world challenges more effectively (63).

### 4.3 Mental health literacy

The literature increasingly recognizes mental health literacy as a crucial component of overall health literacy (8, 64, 65). Mental health literacy encompasses the ability to recognize, manage, and prevent mental health disorders, which is especially critical during adolescence, a peak period for the onset of mental health issues. Educational programs that explicitly focus on mental health literacy, such as those teaching about depression recognition and coping mechanisms, have demonstrated success in reducing stigma and improving help-seeking behaviors among adolescents (66–71). School-based mental health interventions are increasingly recognized for their crucial role in promoting mental health literacy and well-being among students. Collectively, these studies underscore the multifaceted approaches and significant benefits of school-based mental health interventions, advocating for their widespread implementation to foster better mental health outcomes for young individuals (72–76).

### 4.4 Impact of social media and peer education

Social media and peer education represent two pivotal areas in the dissemination of health literacy. Social media platforms extend the reach of health information campaigns and enable peer-to-peer learning dynamics that are influential among adolescents (77–81). Similarly, peer education programs leverage the influence of adolescent social networks to promote healthy behaviors and literacy (82–87). These programs, when well-designed, harness the credibility that peers hold among their groups, significantly impacting attitudes toward health behaviors such as smoking and exercise (88–98). Overall, the literature underscores the transformative potential of digital technologies, interactive learning, mental health literacy, and peer-led initiatives in enhancing health literacy among adolescents. These findings suggest a multifaceted approach to health literacy education, where traditional educational methods are supplemented with innovative strategies that align with the interests and behaviors of present-day youth. Such interventions not only provide personalized health information but also engage users in active learning through interactive features, which have been shown to improve knowledge retention and self-care behaviors significantly.

## 5 Discussion

### 5.1 Implications of the findings

The findings from this review reveal several key implications for the design and implementation of health literacy programs aimed at adolescents. First, the effective use of digital technologies suggests a shift toward more interactive and technology-driven health education. Given the ubiquity of smartphones and Internet access among adolescents, digital platforms offer a valuable medium for delivering personalized, engaging, and timely health information (99). Interactive learning methodologies such as simulations and role-playing can significantly enhance understanding and retention of health information by making learning contextual, practical, and relatable (100).

The emphasis on mental health literacy reflects an urgent need to integrate mental health education into broader health literacy frameworks. This integration is crucial not only for improving adolescents’ ability to manage and prevent mental health issues but also for reducing the stigma associated with these conditions (101, 102, 114). Implementing comprehensive mental health literacy programs within schools could play a pivotal role in early identification and intervention, potentially altering life trajectories in a positive manner (103).

The role of social media and peer education highlights the importance of leveraging social networks and peer influence in health literacy interventions. These platforms and approaches can be particularly effective in spreading health-related messages and fostering normative behavior change, especially in areas where adolescents may feel more comfortable receiving and discussing information among peers (104, 105).

The role of family dynamics in shaping adolescent health literacy is crucial. Parents and guardians not only influence health behaviors but also mediate the access and use of digital tools for health education. Integrating health literacy tools into family settings can enhance communication about health and encourage collective family engagement in healthy behaviors. Programs designed to improve health literacy should, therefore, include components that facilitate parent–child interaction over digital platforms, ensuring that health literacy development is a collaborative family effort (106).

Furthermore, socioeconomic factors significantly influence health literacy levels among adolescents. Disparities in access to digital resources can exacerbate existing inequalities, limiting the effectiveness of digital health literacy interventions. To address this, health education programs must incorporate strategies that are accessible across diverse socioeconomic groups. This includes providing low-cost, high-reach digital solutions and ensuring that health literacy resources are available in multiple formats to cater to varying levels of digital access and literacy (107, 108).

### 5.2 Limitations of the reviewed studies

While the reviewed studies provide valuable insights, they also exhibit several limitations that should be considered. Many studies rely on self-reported data, which can introduce bias and may not accurately reflect actual health literacy levels or behaviors. There is also a lack of longitudinal studies that examine the long-term impacts of health literacy interventions, making it difficult to assess the sustainability of outcomes. The diversity of study designs and measurement tools across the research also poses challenges in comparing results and drawing generalizable conclusions. Many studies do not account for cultural, socioeconomic, and geographic variables that can significantly influence health literacy and behavior among adolescents.

While digital tools offer innovative ways to enhance health literacy, they also present challenges, such as the spread of misinformation and the risk of widening the digital divide. As adolescents increasingly turn to online sources for health information, they may encounter unreliable or misleading content. It is crucial, therefore, for health literacy programs to include critical thinking skills as part of their curriculum, teaching adolescents to evaluate the credibility of online information critically. Interventions should also be designed to be inclusive, ensuring that disadvantaged populations are not left behind in the digital health literacy landscape.

### 5.3 Contributions to the existing literature

This review contributes to the existing literature by synthesizing and highlighting the multifaceted nature of health literacy among adolescents. It expands the understanding of how digital tools and interactive learning environments can be effectively utilized to enhance health literacy. The review also brings mental health literacy to the forefront, advocating for its inclusion as a standard part of health education curricula. By integrating insights from both traditional and innovative educational practices, this review provides a comprehensive perspective on the current state of adolescent health literacy and suggests practical directions for future research and program development.

Overall, this discussion underscores the complexity of health literacy education for adolescents and suggests that a multidimensional approach, which incorporates digital innovation, interactive learning, and peer influence, is likely to be most effective in meeting the health education needs of this demographic. By addressing these aspects, stakeholders can better equip adolescents with the necessary skills to navigate their health throughout their lives.

## 6 Future directions for research

### 6.1 Priority areas for future research

The evolving landscape of adolescent health literacy suggests several key areas for future research. One critical area is the longitudinal evaluation of health literacy interventions to assess their long-term effectiveness and sustainability. This approach would provide valuable insights into how changes in health literacy impact health behaviors and outcomes over time.

Further research is also needed to explore the differential impacts of health literacy interventions across diverse adolescent populations. Studies should consider variables such as socioeconomic status, cultural background, and existing health conditions, which can influence the effectiveness of different health literacy strategies. There is a need to investigate the integration of health literacy into broader educational curricula, examining how subjects such as science, physical education, and social studies can be leveraged to enhance health literacy.

Another important research area involves the development and validation of tools to measure health literacy more accurately and comprehensively among adolescents. This includes the creation of culturally sensitive and age-appropriate assessment tools that can capture the nuances of health literacy in a digital age.

### 6.2 Potential for innovative interventions

The findings from this review also highlight the potential for innovative interventions that utilize new technologies and methodologies to enhance health literacy among adolescents. For example, gamification and virtual reality offer exciting opportunities for creating engaging and immersive learning experiences that could significantly improve understanding and retention of health information.

Social media platforms represent another fertile ground for health literacy interventions. Future interventions could harness these platforms to deliver tailored health messages, facilitate peer-to-peer education, and create communities of practice that encourage healthy behaviors. There is potential to explore how machine learning and artificial intelligence could be employed to personalize health education content based on individual learning styles and preferences.

The influence of community and social networks on adolescent health literacy is profound. Community-based health literacy programs that leverage these networks can enhance the reach and effectiveness of interventions. For example, integrating health literacy projects within community centers and schools can create supportive environments that reinforce the lessons learned through digital and interactive methods. By involving community leaders and peers in these initiatives, programs can harness the inherent trust and communication patterns within these networks, leading to more sustainable health behavior changes.

Innovative interventions could also look at family-based or community-driven health literacy programs that involve parents, caregivers, and local community members. These programs could help reinforce health literacy skills taught in schools and create supportive environments for adolescents to practice and apply these skills in real life.

The integration of mental health literacy into general health education programs is another area ripe for innovative approaches. Programs that address mental health stigma teach coping and resilience skills and provide resources for mental health support need to be developed and tested for their effectiveness within school and community settings.

To ensure the effectiveness of health literacy interventions, robust evaluation methods are essential. These methods should not only assess immediate outcomes but also track long-term behavior changes and health outcomes. For instance, incorporating longitudinal studies into program evaluations can provide insights into the persistence of health literacy skills and their impact over time. Moreover, using a mix of qualitative and quantitative assessment tools can help in understanding both the depth and breadth of the impact of these interventions, facilitating continuous improvement and adaptation.

Addressing the digital divide is critical in ensuring that health literacy interventions do not inadvertently exclude underprivileged segments of the adolescent population. Strategies to mitigate this divide include offering multilingual content, ensuring that digital tools are accessible on low-cost mobile platforms, and providing offline access to critical health literacy content. Creating inclusive content that reflects the diverse cultural, socioeconomic, and personal contexts of adolescents can promote greater engagement and relevance of health literacy tools.

Overall, the future of research in adolescent health literacy should aim not only to fill the gaps identified in current literature but also to innovate and test new methods that can adapt to the changing ways adolescents interact with information and technology. By addressing these areas, researchers and practitioners can better equip future generations with the necessary tools to manage their health effectively.

## 7 Conclusion

### 7.1 Summary of key findings

This review has illuminated several key aspects of health literacy among adolescents, emphasizing the crucial role that emerging technologies and interactive methodologies play in enhancing educational outcomes. The integration of digital tools has proven especially effective, offering personalized and accessible health information that resonates with the tech-savvy nature of today’s youth. Interactive learning methodologies such as simulations and role-playing have been shown to significantly increase engagement and retention of health-related knowledge, providing practical skills that adolescents can apply in real-life contexts.

Mental health literacy emerged as a critical component, with educational initiatives that include mental health components demonstrating effectiveness in reducing stigma and improving help-seeking behaviors among adolescents. The impact of social media and peer education highlights the power of peer influence and the potential of these platforms to disseminate health information effectively and foster healthy behavioral changes.

### 7.2 Recommendations for evidence-based practices

Based on the findings of this review, the following recommendations for evidence-based practices in adolescent health literacy are outlined:

1. Health education programs should continue to leverage digital tools to deliver health information. These tools should be designed to be interactive and user-friendly to keep pace with the technological aptitudes of adolescents.
2. Schools and health educators should incorporate more interactive methodologies into their programs. These should include problem-solving activities, simulations, and role-playing, which help solidify knowledge through practical application.
3. Given the importance of mental health literacy, it is recommended that mental health education be integrated into the broader health literacy curriculum. This integration should include information on recognizing symptoms, effective communication about mental health issues, and strategies for managing mental health.
4. To capitalize on the extensive reach and influence of social media among adolescents, health programs should include components that use these platforms for health education. This involves not only disseminating information but also engaging users in discussions and peer-led activities that promote health literacy.
5. There is a need for the development of comprehensive, culturally appropriate assessment tools to measure health literacy among adolescents more effectively. These tools should be capable of assessing the different dimensions of health literacy, including the ability to obtain, understand, and use health information.
6. Health literacy initiatives should extend beyond the classroom to involve families and communities. This broader engagement can reinforce learning and provide a supportive environment for adolescents to practice their health literacy skills.

By following these recommendations, stakeholders in adolescent health can develop more effective programs that not only improve health literacy but also empower young individuals to make informed health decisions. As our understanding of the best practices in health literacy advances, continuous evaluation and adaptation of these recommendations will be crucial to their success, ensuring they remain relevant and impactful in a rapidly changing world.
